# Supratherapeutic Inhaled Corticosteroid Use in Patients Initiating on Biologic Therapies for Severe Asthma: A Nationwide Cohort Study

**DOI:** 10.1007/s00408-025-00796-5

**Published:** 2025-03-11

**Authors:** Frederikke Hjortdahl, Marianne Baastrup Soendergaard, Susanne Hansen, Anne-Sofie Bjerrum, Anna von Bülow, Ole Hilberg, Barbara Bonnesen, Claus Rikard Johnsen, Sofie Lock Johansson, Linda Makowska Rasmussen, Johannes Martin Schmid, Charlotte Suppli Ulrik, Anne Byriel Walls, Celeste Porsbjerg, Kjell Erik Julius Håkansson

**Affiliations:** 1 Department of Respiratory Medicine, Copenhagen University Hospital, Bispebjerg, Copenhagen, Denmark; 2 Centre for Clinical Research and Prevention, Frederiksberg Hospital, Copenhagen, Denmark; 3 Department of Respiratory Diseases and Allergy, Aarhus University Hospital, Aarhus, Denmark; 4 Sygehus Lillebælt, Vejle Sygehus, Vejle, Denmark; 5 Department of Respiratory Medicine, Gentofte University Hospital, Hellerup, Denmark; 6 Allergy Clinic, Gentofte University Hospital, Hellerup, Denmark; 7 Department of Respiratory Medicine, Odense University Hospital, Odense, Denmark; 8 Department of Respiratory Medicine, Copenhagen University Hospital, Hvidovre, Denmark; 9 Department of Drug Design and Pharmacology, Faculty of Health and Medical Sciences, University of Copenhagen, Copenhagen, Denmark; 10 Capital Region Hospital Pharmacy, Rigshospitalet, Copenhagen, Denmark

**Keywords:** High dose, Adherence, Reduction, Systemic, Oral, Comorbidities, Prospective study

## Abstract

**Background:**

In severe asthma, intensive (“*supratherapeutic*”) doses of inhaled corticosteroids (ICS) are often used. The prevalence of supratherapeutic ICS use and its impact on corticosteroid-related comorbidities is poorly understood. We aimed to describe the prevalence of supratherapeutic ICS use in severe asthma, its relation to corticosteroid-related comorbidities, and changes in prescribed and redeemed ICS dose after 12 months of biologic therapy.

**Methods:**

Patients from the nationwide Danish Severe Asthma Register (DSAR) receiving biologic therapy > 12 months were included. Supratherapeutic doses were defined as > 1600 µg *budesonide* daily. Baseline characteristics, comorbidity burden, and change in ICS use after 12 months of biologic therapy was stratified according to ICS use at baseline.

**Results:**

We included 652 patients in our analyses and 156 (24%) were supratherapeutic ICS users prior to initiation of biologic therapy. Supratherapeutic ICS users had a higher baseline prevalence of cataracts at 14 *vs* 8.1%; *p* = 0.025. No differences in other corticosteroid-related comorbidities were observed.

No change in prevalence of prescribed supratherapeutic ICS was seen after 12 months of biologic therapy. However, a reduction in ICS adherence among supratherapeutic users was observed with 72% of patients demonstrating > 80% adherence at 12 months, compared to 83% at baseline (*p* < 0.001).

**Conclusion:**

Supratherapeutic doses of ICS were used by almost one-fourth of the patients prior to initiation of biologic therapy and were associated with a higher prevalence of cataracts. Physician-driven ICS reduction was rare, yet supratherapeutic ICS users were found to self-regulate ICS therapy when treated with biologic therapy.

**Supplementary Information:**

The online version contains supplementary material available at 10.1007/s00408-025-00796-5.

## Introduction

Severe asthma is a heterogeneous disease seen in approximately 5–10% of the asthma population [1], representing a significant proportion of the morbidity burden, driven by high healthcare resource utilization, loss of productivity, daily symptoms, and poor quality of life due to frequent exacerbations and loss of lung function [2, 3]. For the most severe cases, treatment may include both inhaled corticosteroid (ICS) dosing above recommended daily doses and/or escalation to maintenance oral corticosteroids (mOCS) aiming to achieve disease control [4, 5], despite the availability of biologic therapies for severe asthma [6].

ICS is generally considered to have a flat dose–response curve, with the principal therapeutic benefits seen at low-to-moderate daily doses [7–9]. However, not all patients are well controlled on low-to-medium ICS doses. High-dose ICS use is disease defining for severe asthma [5], and with the increased doses comes an increased risk of both local and systemic adverse effects [10–13]. Previous studies have found that the prevalence of high-dose ICS in real-world populations ranges from 3.1 to 30% depending on age and setting [14–16]. While no official consensus exists, supratherapeutic doses of ICS can be defined as daily exposure to doses above the maximal recommended dose, e.g., *budesonide*-equivalent doses of > 800 or > 1600 µg/day depending on the guideline used [4, 5]. Evidence suggests that high-to-supratherapeutic doses of ICS (> 800–1600 and > 1600 μg/day, respectively) are likely to supress the hypothalamic–pituitary–adrenal (HPA)-axis on par with low dose of oral corticosteroids (OCS) [17]. The association between OCS use and corticosteroid-related comorbidities (e.g., adrenal insufficiency, osteoporosis, type 2 diabetes, depression, cardiovascular diseases, cataracts, and others), even at low daily doses, in asthma is well-established [18–20]. However, less is known about the prevalence of supratherapeutic ICS use in severe asthma, its association with corticosteroid-related comorbidities, and whether these patients are less likely to achieve a significant clinical response to biologic therapy.

The aim of this nationwide cohort study comprising all Danish severe asthma patients initiating biologic therapy in Denmark was to explore the prevalence of supratherapeutic ICS use, investigate the associations between supratherapeutic ICS use and common corticosteroid-related comorbidities, and evaluate the changes in supratherapeutic ICS use after 12 months of biologic therapy.

## Methods

### The Danish Severe Asthma Registry

The current study utilized the Danish Severe Asthma Register (DSAR), a nationwide registry consisting of all Danish patients treated with biologic therapy for severe asthma [21]. DSAR follows a clinical protocol with baseline information collected before the initiation of biologic therapy and follow-up data collected after 4 and 12 months of therapy. All clinics administering biologic therapy in Denmark use the DSAR electronic patient record form.

### Study Approvals and Ethics

All patients provided written consent for their data to be used for research. DSAR was approved by the Capital Region of Copenhagen’s Knowledge Centre for Data Review (VD-2018–31). Patients or the public were not involved in the design of the present study.

### Study Design and Data Sources

The present study utilizes the universal, individual-level linkage between registries made possible by the Danish central person registry. Prescription data were sourced from the National Prescription Registry containing all redeemed prescriptions from 1995 until 2022. Diagnoses of comorbidities was sourced from the National Patient Registry, containing all ICD-10 codes for secondary care contacts from 1994 and onwards.

### Inhaled Corticosteroid Use

Inhaled corticosteroid (ICS) use was assessed on an annual, retrospective basis from 1995 and until the date of first administration of a biologic therapy (“*index date*”). Impact of biologic therapy on ICS use was assessed prospectively based on all redemptions filled within a year from the index date. Annual daily exposure was calculated as the sum of all redeemed doses divided by 365. Adherence was defined as redeemed daily exposure divided by prescribed daily dose, and > 80% was defined as acceptable adherence. DSAR currently does not employ a standardized ICS downtitration protocol, and prescribed ICS reductions are performed as deemed indicated by the treating physician.

ICS dose-equivalency was calculated using budesonide-equivalent doses as previously described [14]. ICS dose categories were assigned according to the Global Initiative for Asthma (GINA) 2023 guidelines [5], and supratherapeutic ICS use was defined as > 1600 µg *budesonide* equivalents daily.

### Comorbidities

Comorbidities were assessed using two strategies:

1. Physician-assessed severe asthma-related comorbidities at baseline according to the DSAR protocol [21]. Assessment is based on journal records, prescribed medications, patient-report, and clinical examination at the baseline visit.
2. For corticosteroid-related comorbidities, ATC-codes for prescriptions from 1995 and onward and ICD-10-codes for secondary care diagnoses were identified 10 years prior to the index date. Comorbidities and definitions used are previously described [22, 23] and are found in Supplementary Table 1. The combined use of prescriptions and secondary care diagnoses allows for capture of both primary care treated and secondary care managed comorbidities.

### Sensitivity Analyses

Two sensitivity analyses were performed. First, changes in ICS use were stratified according to biologic treatment response after 12 months were investigated. Second, differences in response to biologic therapy according to prescribed ICS dose at baseline were investigated.

Response was described by two outcomes: clinical response and remission, as previously outlined [23]. The definition of clinical response was based on the criterion setting the indication for initiating biologic therapy (exacerbations and/or the need for mOCS). Hence, clinical response can be defined as (1) a reduction of at least 50% in the annualized exacerbation rate, and (2) a reduction of at least 50% in the mOCS dose. Clinical remission was characterized by the complete absence of exacerbations and mOCS use, as well as absence of uncontrolled symptoms (an Asthma Control Questionnaire (ACQ-6) score ≤ 1.5) and a normal lung function (forced expiratory volume in one second (FEV_1_) of at least 80% of the predicted).

### Statistical Analyses

Descriptive variables, ICS use, and comorbidity burden are presented as n (%) or median (interquartile range). Between-group differences were investigated using either Wilcoxon rank-sum test, Chi-squared or Fisher’s exact test depending on data type. R 4.3.1 (The R Foundation, AU) was used for statistical analyses. Statistical significance was set at *p* ≤ 0.05. Ggplot2 and BioRender were used for graphics and plots.

## Results

**Fig. 1:**
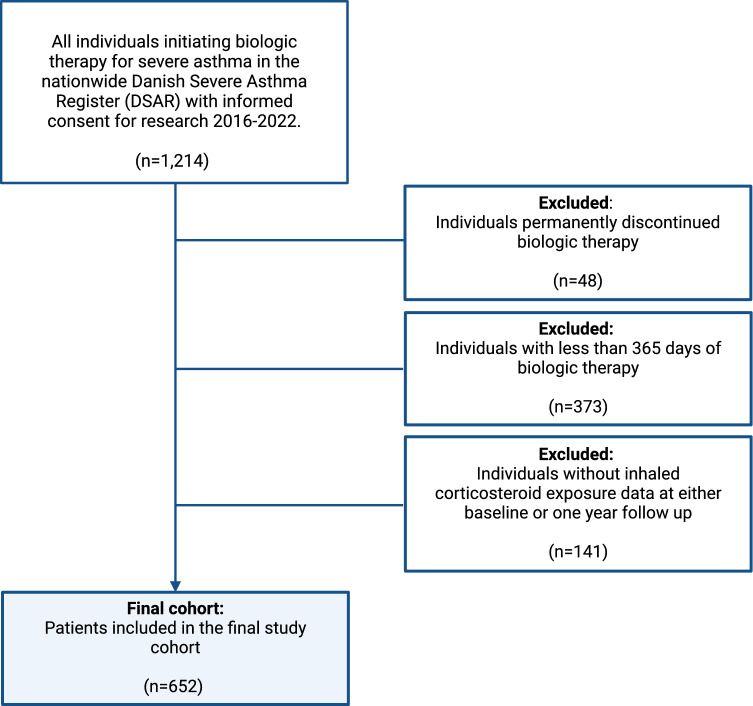
Inclusion flow in a nationwide cohort of 652 patients with severe asthma eligible for biologic therapy, categorized as supratherapeutic ICS users and non-supratherapeutic ICS users

Of the 1214 severe asthma patients initiated on biologic therapy in DSAR who had given informed consent for research, patients who had completed less than 12 months of biologic therapy and patients without ICS exposure data at either baseline or 12-month follow-up were excluded, and 652 patients were included in the present study (Fig. 1).

### Prevalence of Supratherapeutic ICS Use and Comorbidity Burden

When stratified according to daily ICS exposure, 156 patients (24%) were classified as supratherapeutic ICS users, and 496 patients (76%) were classified as non-supratherapeutic ICS users. Supratherapeutic ICS users and non-supratherapeutic ICS users were similar in age, sex, BMI, smoking status, duration of disease, inflammatory biomarkers, and lung function at baseline. Patient-reported outcomes such as the Asthma Control Questionnaire were similar between supratherapeutic ICS users and non-users at baseline, but supratherapeutic ICS users tended to have a more exacerbations at baseline (3 (2–4) *vs* 2 (1–4), *p* = 0.058) (Table 1).

**Table 1:** Baseline characteristics of supratherapeutic ICS users *vs* non-supratherapeutic ICS users from a nationwide cohort of 652 patients with severe asthma initiating biologic therapy

	Overall (*n* = 652)	Supratherapeutic ICS users (*n* = 156)	Non-supratherapeutic ICS users (*n* = 496)	p-value^a^
Age (years)	55 (46–65) *n* = 652	55 (47–64) *n* = 156	56 (46–65) *n* = 496	> 0.9
Female	347 (53%) *n* = 652	87 (56%) *n* = 156	260 (52%) *n* = 496	0.5
BMI (kg·m^−2^)	26.8 (23.9–31.0) *n* = 640	26.7 (23.9–31.2) *n* = 155	26.8 (23.9–30.9) *n* = 485	> 0.9
*Smoking status*				0.8
Current smoker	17 (2.7%) *n* = 638	5 (3.2%) *n* = 154	12 (2.5%) *n* = 484	
Ex-smoker	286 (45%) *n* = 638	70 (45%) *n* = 154	216 (45%) *n* = 484	
Never-smoker	335 (53%) *n* = 638	79 (51%) *n* = 154	256 (53%) *n* = 484	
Pack-years	13 (6–25) *n* = 287	15 (8–26) *n* = 73	12 (5–24) *n* = 214	0.2
Duration of disease (years)	20 (6–35) *n* = 403	17 (6–31) *n* = 105	21 (7–36) *n* = 298	0.4
*Age at onset*				0.2
Childhood (< 18)	176 (40%) *n* = 438	36 (33%) *n* = 110	140 (43%) *n* = 328	
Early (18–40)	99 (23%) *n* = 438	27 (25%) *n* = 110	72 (22%) *n* = 328	
Late (> 40)	163 (37%) *n* = 438	47 (43%) *n* = 110	116 (35%) *n* = 328	
*Inflammatory biomarkers & allergy testing*				
Blood eosinophils ($\times$ 10^9^ L^−1^)	0.39 (0.17–0.65) *n* = 510	0.39 (0.17–0.61) *n* = 130	0.38 (0.17–0.66) *n* = 380	> 0.9
F_E_NO (ppb)	30 (17–57) *n* = 440	29 (18–55) *n* = 118	30 (16–58) *n* = 322	0.8
Total IgE (IU $\times$ mL^−1^)	154 (63–395) *n* = 367	130 (40–381) *n* = 106	166 (73–399) *n* = 261	0.14
Positive allergy test	48 (69%) *n* = 70	15 (65%) *n* = 23	33 (70%) *n* = 47	0.7
*Lung function*				
FEV_1_ (L)	2.15 (1.60–2.79) *n* = 567	2.04 (1.53–2.76) *n* = 141	2.17 (1.62–2.81) *n* = 426	0.4
FEV_1_ (% pred)	69 (56–83) *n* = 555	67 (53–86) *n* = 140	69 (56–83) *n* = 415	0.6
FEV_1_/FVC	0.66 (0.57–0.74) *n* = 547	0.65 (0.56–0.74) *n* = 137	0.66 (0.57–0.74) *n* = 410	0.4
Patient-reported outcomes				
*Asthma Control Questionnaire*				
At baseline	2.50 (1.52–3.17) *n* = 346	2.57 (1.33–3.40) *n* = 101	2.50 (1.57–3.17) *n* = 245	0.4
At 12 months	1.14 (0.50–2.00) *n* = 365	1.14 (0.57–2.50) *n* = 101	1.14 (0.50–1.86) *n* = 264	0.2
*Asthma Quality of Life Questionnaire*				
At baseline	20.0 (17.8–22.9) *n* = 95	20.5 (19.0–22.6) *n* = 20	19.6 (17.7–22.9) *n* = 75	0.5
At 12 months	25.2 (22.3–26.6) *n* = 178	24.1 (22.0–26.5) *n* = 39	25.3 (22.4–26.6) *n* = 139	0.3
*Sinonasal Outcome Test 22*				
At baseline	34 (21–48) *n* = 173	35 (22–53) *n* = 45	34 (21–47) *n* = 128	0.7
At 12 months	22 (11–34) *n* = 245	24 (11–33) *n* = 65	22 (11–34) *n* = 180	0.8
*Annual exacerbation rate*				
At baseline	2 (1–4) *n* = 513	3 (2–4) *n* = 130	2 (1–4) *n* = 383	0.058
At 12 months	0 (0–1) *n* = 428	0 (0–1) *n* = 109	0 (0–1) *n* = 319	0.6

Data are presented as n (%) or median (interquartile range) unless otherwise is stated. ICS: inhaled corticosteroid; BMI: body mass index; F_E_NO: exhaled nitric oxide fraction; IgE: Immunoglobulin E; FEV_1_: forced expiratory volume in 1 s; FVC: forced vital capacity

^a^Wilcoxon rank-sum test, Pearson’s Chi-squared test or Fisher’s exact test used

Among physician-assessed severe asthma-related comorbidities, diabetes was more prevalent in supratherapeutic ICS users (15 *vs* 8.8%, *p* = 0.035). Aspirin-exacerbated respiratory disease (AERD) was less prevalent in supratherapeutic ICS users (3.9 *vs* 9.4%; *p* = 0.031) compared to non-supratherapeutic users. A trend toward gastroesophageal reflux disease (GERD) being more prevalent in supratherapeutic ICS users (38 *vs* 30%; *p* = 0.071) was observed. In terms of corticosteroid-related comorbidities, the baseline prevalence of cataract was significantly higher among supratherapeutic ICS users compared to non-supratherapeutic ICS users (14 *vs* 8.1%; *p* = 0.025). However, we did not observe any other differences in prevalences of other corticosteroid-related comorbidities between supratherapeutic- and non-supratherapeutic ICS users (Table 2). In a sensitivity analysis restricted to patients prescribed supratherapeutic ICS stratified by ICS adherence, no differences in number of or specific corticosteroid-related comorbidities were found (Supplementary Table 2).

**Table 2:** Physician-assessed severe asthma-related and corticosteroid-related comorbidities in supratherapeutic ICS users *vs* non-supratherapeutic ICS users from a nationwide cohort of 652 patients with severe asthma initiating biologic therapy

	Overall (*n* = 652)	Supratherapeutic ICS users (*n* = 156)	Non-supratherapeutic ICS users (*n* = 496)	p-value^a^
*Physician assessed comorbidities*				
ABPA	21 (3.3%) *n* = 631	6 (3.9%) *n* = 155	15 (3.2%) *n* = 476	0.7
AERD	50 (8.0%) *n* = 623	6 (3.9%) *n* = 153	44 (9.4%) *n* = 470	0.031
Allergic rhinitis	355 (56%) *n* = 633	89 (57%) *n* = 156	266 (56%) *n* = 477	0.8
Atopy	130 (21%) *n* = 628	32 (21%) *n* = 156	98 (21%) *n* = 472	> 0.9
Bronchiectasis	159 (25%) *n* = 633	47 (30%) *n* = 156	112 (23%) *n* = 477	0.10
Cardiovascular disease	190 (30%) *n* = 630	48 (31%) *n* = 155	142 (30%) *n* = 475	0.8
Chronic rhinosinusitis	396 (63%) *n* = 632	92 (59%) *n* = 156	304 (64%) *n* = 476	0.3
COPD	148 (24%) *n* = 629	43 (28%) *n* = 155	105 (22%) *n* = 474	0.2
Diabetes	65 (10%) *n* = 631	23 (15%) n156	42 (8.8%) *n* = 475	0.035
Dysfunctional breathing	47 (7.5%) *n* = 629	13 (8.4%) *n* = 155	34 (7.2%) *n* = 474	0.6
Eczema	163 (26%) *n* = 630	44 (28%) *n* = 156	119 (25%) *n* = 474	0.4
EGPA	24 (3.8%) *n* = 626	5 (3.2%) *n* = 154	19 (4.0%) *n* = 472	0.7
Eosinophilic pneumonia	19 (3.0%) *n* = 627	4 (2.6%) *n* = 155	15 (3.2%) *n* = 472	> 0.9
GERD	198 (32%) *n* = 623	58 (38%) *n* = 154	140 (30%) *n* = 469	0.071
Nasal polyps	267 (42%) *n* = 630	65 (42%) *n* = 154	202 (42%) *n* = 476	> 0.9
OSAS	71 (11%) *n* = 631	18 (12%) *n* = 156	53 (11%) *n* = 475	0.9
Osteoporosis	147 (39%) *n* = 380	39 (43%) *n* = 90	108 (37%) *n* = 290	0.3
Psychiatric disease	87 (14%) *n* = 628	21 (14%) *n* = 155	66 (14%) *n* = 473	0.9
Vocal cord dysfunction	16 (2.6%) *n* = 625	7 (4.5%) *n* = 154	9 (1.9%) *n* = 471	0.082
*Corticosteroid-related comorbidities*				
Any comorbidity	603 (92%)	148 (95%)	455 (92%)	0.2
Number of comorbidities	3 (2–4)	3 (2–4)	3 (2–4)	0.4
Adrenal insufficiency	32 (4.9%)	11 (7.1%)	21 (4.2%)	0.2
Cataracts	62 (9.5%)	22 (14%)	40 (8.1%)	0.025
Glaucoma	4 (0.6%)	0 (0%)	4 (0.8%)	0.6
Cardiovascular diseases	461 (71%)	107 (69%)	354 (71%)	0.5
Diabetes mellitus, Type 2	93 (14%)	28 (18%)	65 (13%)	0.13
Depression or/and anxiety	163 (25%)	38 (24%)	125 (25%)	0.8
Dysphonia	5 (0.8%)	2 (1.3%)	3 (0.6%)	0.3
Fracture	178 (27%)	49 (31%)	129 (26%)	0.2
GERD	412 (63%)	97 (62%)	315 (64%)	0.8
Heart failure	15 (2.3%)	3 (1.9%)	12 (2.4%)	> 0.9
Ischemic heart disease	33 (5.1%)	7 (4.5%)	26 (5.2%)	0.7
Obesity	95 (15%)	24 (15%)	71 (14%)	0.7
Oral candidiasis	153 (23%)	44 (28%)	109 (22%)	0.11
Osteoporosis	211 (32%)	56 (36%)	155 (31%)	0.3
OSAS	66 (10%)	15(9.6%)	51 (10%)	0.8

Data are presented as n (%) or median (IQR), unless otherwise is stated

^a^Wilcoxon rank-sum test, Pearson’s Chi-squared test or Fisher’s exact test used

^b^Based on ATC-codes for prescriptions from 1995 and onward ICD-10-codes for secondary care diagnoses 10 years prior to the index date

*ICS* Inhaled corticosteroid, *ABPA* allergic bronchopulmonary aspergillosis, *AERD* Aspirin-exacerbated respiratory disease, *COPD* chronic obstructive pulmonary disease, *EGPA* eosinophilic granulomatosis with polyangiitis, *GERD* Gastroesophageal reflux disease, *OSAS* Obstructive sleep apnea syndrome

### Prescribed Inhaled Corticosteroid Dose, Biologic Therapy, and Patient Adherence

Supratherapeutic ICS users were exposed to a median daily ICS dose of 2055 (1805–2374) µg *budesonide* equivalent at baseline, whereas non-supratherapeutic ICS users were exposed to a median daily ICS dose of 970 (633–1266) µg *budesonide* equivalents. Supratherapeutic ICS users had a higher baseline adherence to ICS (105% of daily prescribed doses redeemed (88–128%) *vs* 66% (46–91%); *p* < 0.001) compared to non-supratherapeutic ICS users. Furthermore, 83% of patients prescribed supratherapeutic ICS had an acceptable adherence to ICS therapy at baseline compared to 37% of patients prescribed non-supratherapeutic doses. Additionally, we observed a trend toward slightly more supratherapeutic ICS users being on mOCS at baseline (31 *vs* 24%; *p* = 0.077) (Table 2). Retrospective ICS exposure data demonstrate that while median ICS dose exposure seems to increase over time (Supplementary Fig. 1A), a significant portion of patients have long-term exposure to supratherapeutic ICS (Supplementary Fig. 1B).

**Fig. 2:**
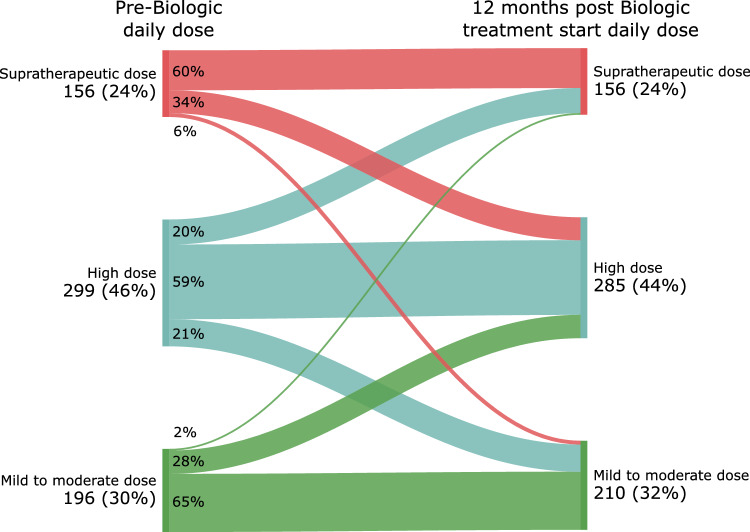
Sankey plot describing the impact of 12 months with biologic therapy according to change in exposed (adherence-driven) daily dose of ICS, categorized by either supratherapeutic, high and mild-to-moderate dose

The change in exposed (adherence-driven) daily dose of ICS after treatment with biologic therapy is illustrated in Fig. 2. Irrespective of baseline ICS exposure, most patients did not change in daily ICS exposure after 12 months of therapy. Transitions between exposure groups were seen in smaller portions, with 34% of supratherapeutic ICS users down-titrating to high-dose and 20% of high-dose users up-titrating to supratherapeutic doses, resulting in an overall unchanged prevalence of 24% (Fig. 2). Of note, a slight decrease (1600 (1600–2400) µg) in the median prescribed ICS dose after 12 months was observed in those using supratherapeutic ICS doses at baseline (Table 3).

**Table 3:** Background treatment and clinical treatment outcomes 12 months post-biologic therapy of supratherapeutic ICS users *vs* non-supratherapeutic ICS from a nationwide cohort of 652 patients with severe asthma initiating biologic therapy

	Overall (*n* = 652)	Supratherapeutic ICS users (*n* = 156)	Non-supratherapeutic ICS users (*n* = 496)	p-value^b^
*Daily exposed dose of ICS (µg)*^*a*^				
At baseline	1174 (725–1582) *n* = 652	2055 (1805–2374) *n* = 156	970 (633–1266) *n* = 496	< 0.001
At 12 months	1157 (658–1578) *n* = 652	1782 (1447–2362) *n* = 156	985 (579–1315) *n* = 496	< 0.001
*Daily prescribed dose of ICS (µg)*^a^				
At baseline	1600 (1200–1600) *n* = 490	2000 (1600–2400) *n* = 118	1600 (800–1600) *n* = 372	< 0.001
At 12 months	1600 (800–1600) *n* = 470	1600 (1600–2400) *n* = 112	1600 (800–1600) *n* = 358	< 0.001
*Daily prescribed dose of mOCS (mg)*				
Proportion on mOCS at baseline (n)	167 (26%) *n* = 649	48 (31%) *n* = 154	119 (24%) *n* = 495	0.077
At baseline (mg)	10 (5–13) *n* = 135	10 (8–13) *n* = 39	8 (5–10) *n* = 96	0.15
At 12 months (mg)	7.5 (5.0–10.0) *n* = 63	7.5 (5.0–10.0) *n* = 19	7.5 (5.0–10.0) *n* = 44	0.9
*Patient adherence to ICS (%)*				
At baseline	79 (53–99) *n* = 490	105 (88–128) *n* = 118	66 (46–91) *n* = 372	< 0.001
At 12 months	79 (58–101) *n* = 470	96 (78–115) *n* = 112	74 (49–99) *n* = 358	< 0.001
*Acceptable adherence to ICS (> 80%) (n)*				
At baseline	236 (48%) *n* = 490	98 (83%) *n* = 118	138 (37%) *n* = 372	< 0.001
At 12 months	225 (48%) *n* = 470	81 (72%) *n* = 112	144 (40%) *n* = 358	< 0.001
*Additional controllers at baseline*				
Long-acting muscarinic antagonists	240 (37%) *n* = 649	67 (44%) *n* = 154	173 (35%) *n* = 495	0.055
Long-acting ${\upbeta }_{2}$-agonists	457 (70%) *n* = 649	115 (75%) *n* = 154	342 (69%) *n* = 495	0.2
Dual long-acting bronchodilators	221 (34%) *n* = 649	63 (41%) *n* = 154	158 (32%) *n* = 495	0.040
Short acting ${\upbeta }_{2}$-agonists	328 (51%) *n* = 649	76 (49%) *n* = 154	252 (51%) *n* = 495	0.7
Leukotriene receptor antagonists	238 (37%) *n* = 649	61 (40%) *n* = 154	177 (36%) *n* = 495	0.4
Theophylline	22 (3.4%) *n* = 649	10 (6.5%) *n* = 154	12 (2.4%) *n* = 495	0.015
Azithromycin	11 (1.7%) *n* = 649	2 (1.3%) *n* = 154	9 (1.8%) *n* = 495	> 0.9
*Response to biologic therapy*				> 0.9
Non-response	42 (12%) *n* = 361	12 (12%) *n* = 103	30 (12%) *n* = 258	
Clinical response	247 (68%) *n* = 361	69 (67%) *n* = 103	178 (69%) *n* = 258	
Clinical remission	72 (20%) *n* = 361	22 (21%) *n* = 103	50 (19%) *n* = 258	

Data are presented as n (%) or median (IQR) unless otherwise is stated. ICS: inhaled corticosteroid; mOCS: maintenance oral corticosteroid. Data are presented as: n (%) or median (IQR) unless otherwise is stated. ICS: inhaled corticosteroid; mOCS: maintenance oral corticosteroid

^a^budesonide-equivalent dose

^b^Wilcoxon rank-sum test, Pearson’s Chi-squared test or Fisher’s exact test used

### Clinical Response and Remission

When stratified according to treatment response after 12 months of biologic therapy, no differences in proportions of patients achieving clinical response and/or remission were observed between supratherapeutic ICS users and non-supratherapeutic ICS users (Table 3). Of those achieving clinical remission after 12 months of biologic therapy, 28% of patients continued to be exposed to supratherapeutic ICS doses (Supplementary Table 3).

## Discussion

In this nationwide study of 652 individuals with severe asthma initiating biologic therapy, supratherapeutic ICS use was observed in 24% of patients. Supratherapeutic ICS users had higher prevalence of cataracts at baseline; however, other corticosteroid-related comorbidities, clinical biomarkers, and lung function parameters were comparable to non-supratherapeutic ICS users. After 12 months of biologic therapy, no change in the prevalence of supratherapeutic ICS use was observed. Finally, no differences in response and remission rates after 12 months of biologic therapy between supratherapeutic ICS users and non-supratherapeutic users were seen.

Evidence regarding the prevalence of supratherapeutic ICS use in patients with severe asthma is sparse. In a nationwide cohort study of Swedish patients with severe asthma, 8.6% were treated with a median daily ICS dose of 2192 µg *budesonide* equivalents, indicating that a significant fraction of patients are treated with supratherapeutic ICS doses [14]. In terms of real-world ICS exposure, Danish cohort studies found high-dose ICS use among children and adolescents aged 2–17 years ranging between 3.1 and 30%, depending on age [16], and in patients aged 18–45 years, the prevalence was 6.2% [15].

As most of the therapeutic benefit of ICS (in outcomes such as FEV_1_, morning and evening peak expiratory flow (PEF) and $\upbeta$-agonist use) in mild-to-moderate asthma is achieved at a low daily dose of approximately 400 µg inhaled *budesonide,* with no additional effects seen above 1000 µg daily [7], and no additional benefit of high-dose ICS treatment has been demonstrated across a range of outcomes such as exacerbation prevention [25, 26], the clinical evidence for supratherapeutic use in severe asthma is at best sparse. Arguably, it is crucial to consider individual variability (e.g., genetic variations) in the dose–response of ICS in patients with asthma [26, 27], and especially in severe asthma as steroid resistance is thought to be more common [28], resulting in the probable need for higher doses of ICS to reduce the frequency of severe acute exacerbations [29–31]. In this study, we found a tendency toward an increased baseline exacerbation rate and mOCS use among supratherapeutic users suggesting a ceiling effect.

All corticosteroids, irrespective of route of administration, are associated with an increased risk of adrenal insufficiency [32]. Further, a daily dose of 1000 µg inhaled *budesonide* appears to be equivalent in causing the same degree of adrenal suppression as 2 mg of *prednisolone* once daily [17], suggesting that supratherapeutic use of ICS equals a low dose of OCS treatment in physiologic effects. As previously mentioned, OCS use is associated with a wide range of corticosteroid-related comorbidities (e.g., adrenal insufficiency, osteoporosis, type 2 diabetes, cardiovascular diseases, depression and cataract), with the risk increasing in a dose-dependent manner [13, 19, 20, 22]. Even a low dose of OCS exposure (i.e., < 5 mg *prednisone* equivalent daily) is suggested to increase the risk of corticosteroid-related complications in patients with severe asthma [18]. Taken together, this suggests that while the reduction of mOCS treatment remains vital in severe asthma, reducing the inhaled corticosteroid burden may also be an important factor in reducing corticosteroid-related morbidity. Indeed, the corticosteroid burden in asthma is suggested to be cumulative both for oral and inhaled corticosteroids [33–35]. Despite this, we found only one significant association between supratherapeutic ICS use and corticosteroid-related comorbidities: cataract, a well-described complication related to high-dose ICS use [13, 36, 37]. However, the entire population of this study had a significant history of OCS exposure, making it difficult to isolate the effects of supratherapeutic ICS exposure on corticosteroid-related comorbidities. Recent studies have associated high-dose ICS exposure to systemic corticosteroid-related comorbidities after taking confounding by OCS into account [34, 35].

The oral steroid-sparing effects of biologic therapy are well-documented, with dose reductions of mOCS ranging between 45 and 75% [38–40]. In terms of ICS, preliminary findings from a subsample of the DSAR, where reduction of ICS treatment did not lead to a higher rate of exacerbations during biologic therapy [41], and a newly published phase 4 study reported, that 92% of patients with severe asthma receiving biologic therapy*,* managed to effectively reduce their high-dose ICS [42]. Additionally, among supratherapeutic ICS users, we saw an overall reduction in adherence to ICS, suggesting that some patients do reduce their exposure without physician intervention. Despite above indications of safety, we found little evidence of physician-driven dose de-escalation overall. This de-escalation inertia may be driven by the relative lack of evidence, the time required for practice change, and a hesitancy from both patients and physicians due to the pre-biologic severity of disease. Hence, a longer timeframe of 24–36 months may reveal a larger incidence of treatment de-escalation of ICS.

In the context of safety and efficacy of supratherapeutic ICS use, we found no differences in the clinical outcomes of biologic therapy itself. While there is no consensus on specific criteria for all domains of remission in severe asthma [24], absence and/or reduced use of mOCS is typically included [43]. Considering that 28% of patients in the present study received supratherapeutic ICS doses after achieving clinical remission, it is debatable whether it is fair to claim remission in patients exposed to ICS doses equivalent to a low daily dose of OCS.

The present study is strengthened by its use of real-world data from severe asthma patients initiated on biologic therapy and its linkage to validated national registries allowing for both retro- and prospective follow-up. Despite this, we acknowledge that there are limitations. First, ICS exposure is based on redemption, not administered doses. Second, we are unable to assess the true corticosteroid-related morbidity induced by supratherapeutic ICS use due to the widespread use of OCS. We must also emphasize the descriptive nature of our data. Although, association between corticosteroid-related comorbidities and supratherapeutic ICS use can be established, we cannot determine causality. In terms of comorbidities, differences in physician-assessed and database-assessed comorbidity prevalence may be attributed to recall- or reporting bias as patient-report is part of the physician assessment. Finally, due to its real-world design, some data will inevitably be missing, which in pre-/post-analyses limits analyses to patients with available data at both times. This may introduce selection bias if patients with missing data are significantly different from those with available data, yet data in DSAR are assumed to be missing at random.

## Conclusion

Supratherapeutic doses of ICS were used by almost one-fourth of the patients prior to initiation of biologic therapy and were associated with a higher prevalence of cataracts. Physician-driven ICS reduction was rare, yet supratherapeutic ICS users were found to self-regulate ICS therapy when treated with biologic therapy.

## Supplementary Information

Below is the link to the electronic supplementary material.

Supplementary Fig. 1. Exposure to inhaled corticosteroids over a 26-year period including baseline of initiating biologic therapy and 1-year post-biologic therapy in severe asthma patients **A** the median daily budesonide-equivalent exposure in mcg **B** the median daily budesonide-equivalent exposure in mcg categorized by either moderate-to-low, high or supratherapeutic dose. Bx: biologic therapy
Supplementary file1 (PDF 9 KB)

Supplementary file2 (DOCX 200 KB)

## Data Availability

No datasets were generated or analyzed during the current study.

## Abbreviations

ICS: Inhaled corticosteroids
DSAR: Danish Severe Asthma Register
mOCS: Maintenance oral corticosteroids
OCS: Oral corticosteroids
GERD: Gastroesophageal reflux disease
AERD: Aspirin-exacerbated respiratory disease
ACQ-6: Asthma control questionnaire 6
FEV_1_: Forced expiratory volume in one second
PEF: Morning and evening peak expiratory flow
