# Supplementary figures and images for: Supratherapeutic Inhaled Corticosteroid Use in Patients Initiating on Biologic Therapies for Severe Asthma: A Nationwide Cohort Study
Source: Lung. 2025 Mar 11;203(1):42. doi: 10.1007/s00408-025-00796-5 (PMC11897081; doi:10.1007/s00408-025-00796-5)

**A**Median (interquartile range) daily budesonide--  
equivalent exposure (mcg)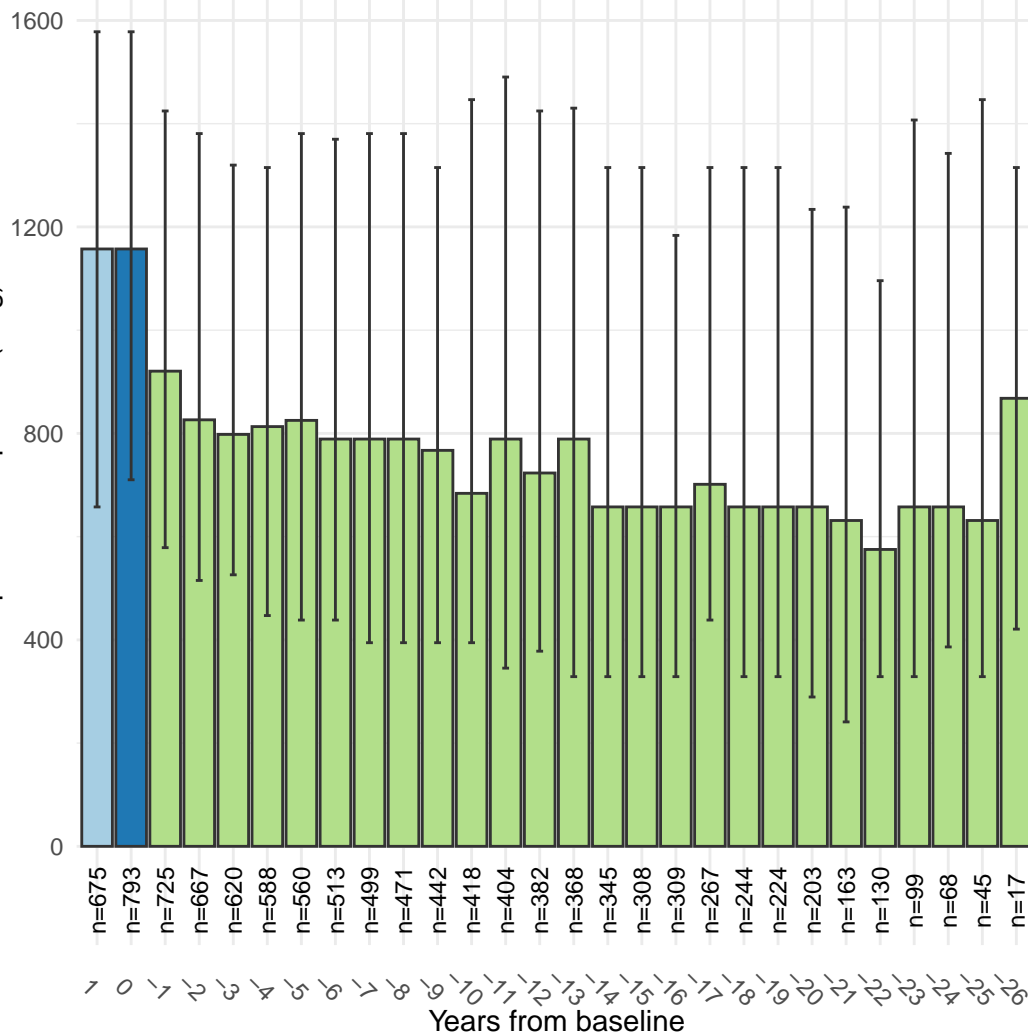**B**

Percent (%)

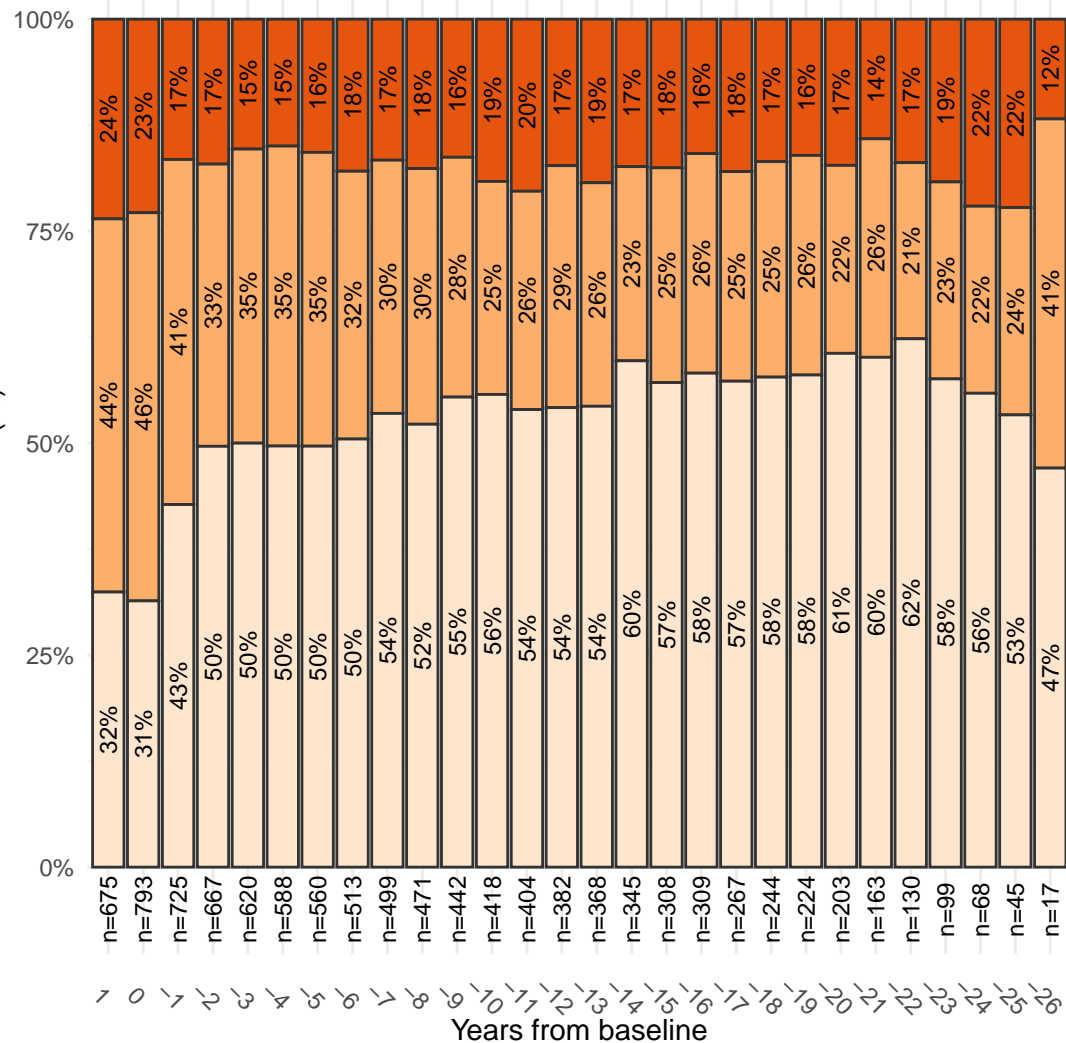

Supplement: Supplementary file 1 — Supplementary Fig. 1. Exposure to inhaled corticosteroids over a 26-year period including baseline of initiating biologic therapy and 1-year post-biologic therapy in severe asthma patients A the median daily budesonide-equivalent exposure in mcg B the median daily budesonide-equivalent exposure in mcg categorized by either moderate-to-low, high or supratherapeutic dose. Bx: biologic therapy Supplementary file1 (PDF 9 KB) [file 408_2025_796_MOESM1_ESM.pdf]
