# Supplementary material for: Supratherapeutic Inhaled Corticosteroid Use in Patients Initiating on Biologic Therapies for Severe Asthma: A Nationwide Cohort Study
Source: Lung. 2025 Mar 11;203(1):42. doi: 10.1007/s00408-025-00796-5 (PMC11897081; doi:10.1007/s00408-025-00796-5)
Supplement: Supplementary file 2 — Supplementary file2 (DOCX 200 KB) [file 408_2025_796_MOESM2_ESM.docx]

**SUPPLEMENTARY TABLES**

**Supratherapeutic inhaled corticosteroid use in patients initiating on biologic therapies for severe asthma – a nationwide study from the Danish Severe Asthma Registry**

**Frederikke Hjortdahl, MSc^1^ ; Marianne Baastrup Soendergaard, MD,PhD^1^; Susanne Hansen, MSc, PhD^1,2^; Anne-Sofie Bjerrum, MD, PhD^3^; Anna von Bülow, MD, PhD^1^; Ole Hilberg, MD, DMSc^4^; Barbara Bonnesen Bertelsen, MD, PhD^5^; Claus Rikard Johnsen, MD^6^; Sofie Lock Johansson, MD, PhD^7^; Linda Makowska Rasmussen, MD, PhD^6^; Johannes Martin Schmid, MD, PhD^3^; Charlotte Suppli Ulrik, MD, DMSc^8^; Anne Byriel Walls^9,10^ PhD; Celeste Porsbjerg, MD, PhD^1^; Kjell Erik Julius Håkansson^1^, MD, PhD**

^1^Dept of Respiratory Medicine, Copenhagen University Hospital – Bispebjerg, Copenhagen, Denmark ^2^Centre for Clinical Research and Prevention, Frederiksberg Hospital, Copenhagen, Denmark ^3^Dept of Respiratory Diseases and Allergy, Aarhus University Hospital, Aarhus, Denmark ^4^Sygehus Lillebælt – Vejle Sygehus, Vejle, Denmark ^5^Dept of Respiratory Medicine, Gentofte University Hospital, Hellerup, Denmark ^6^Allergy Clinic, Gentofte University Hospital, Hellerup, Denmark ^7^Dept of Respiratory Medicine, Odense University Hospital, Odense, Denmark ^8^Dept of Respiratory Medicine, Copenhagen University Hospital – Hvidovre, Denmark ^9^Dept of Drug Design and Pharmacology, Faculty of Health and Medical Sciences, University of Copenhagen, Copenhagen, Denmark ^10^Capital Region Hospital Pharmacy, Rigshospitalet, Copenhagen, Denmark

| Supplementary Table 1. Common corticosteroid-related comorbidities | | |
| --- | --- | --- |
| Comorbidity | **ATC-code** | **ICD-10 code** |
| Osteoporosis | M05, A12A, A11CB, A11CC | M80-M82 |
| Fractures |  | T02, T08, T10, T12, T142, M484, M485, M80, M843, M844, S02 (excl. S025), S12 (excl. S128, S129), S22, S32, S42, S52, S62, S72, S82, S92 |
| Osteonecrosis |  | M87 |
| Diabetes mellitus, Type 2 | A10B | E11 |
| Adrenal insufficiency |  | E273, E274A, E274C |
| Cardiovascular Disease | B01, C | I20-I25, I11.0; I13.0; I13.2; I42.0; I42.6; I42.7; I 42.9; I50.0; I50.1; I50.9 |
| Depression/anxiety | N06AB, N05A | F32-F33; F40-F41 |
| Peptic Ulcers | A02 | K25-28 |
| Cataract |  | H25, H26, H28 |
| Glaucoma |  | H40, H42 |
| Obesity | A08A | E66 |
| Sleep Apnoea |  | G473 |
| Psychiatric Disease | N05A | F31, F2 |
| Oral candidiasis | A01AB09, A07AA02 | DB378C, DB370 |
| Dysphonia |  | DR490 |
| ATC-code: Anatomical Therapeutic Chemical Classification System; ICD-10 code: International Classification of Diseases 10^th^ Revision | | |

| **Supplementary Table 2.** Corticosteroid related comorbidities in patients prescribed supratherapeutic ICS users (adherent) *versus* patients prescribed supratherapeutic ICS users (non-adherent) from a nationwide cohort of 652 patients with severe asthma initiating biologic therapy | | | | |
| --- | --- | --- | --- | --- |
|  | **Overall**  **(n=119)** | **Patients prescribed supratherapeutic ICS, adherent**  **(n=45)** | **Patients prescribed supratherapeutic ICS, non-adherent**  **(n=74)** | **p-value^a^** |
| **Corticosteroid-related comorbidities** | | | | |
| **Any comorbidity** | 109 (92%) | 40 (89%) | 69 (93%) | 0.5 |
| **Number of comorbidities** | 3 (1, 4) | 3 (2,4) | 2 (1,4) | 0.5 |
| Adrenal insufficiency | 14 (12%) | 4 (8.9%) | 10 (14%) | 0.4 |
| Cataracts | 12 (10%) | 6 (13%) | 6 (8.1%) | 0.4 |
| Glaucoma | 0 (0%) | 0 (0%) | 0 (0%) | N/A |
| Cardiovascular diseases | 77 (65%) | 33 (73%) | 44 (59%) | 0.12 |
| Diabetes mellitus, Type 2 | 16 (13%) | 8 (18%) | 8 (11%) | 0.3 |
| Depression or/and anxiety | 28 (24%) | 12 (27%) | 16 (22%) | 0.5 |
| Dysphonia | 0 (0%) | 0 (0%) | 0 (0%) | N/A |
| Fracture | 37 (36%) | 16 (36%) | 27 (36%) | >0.9 |
| GERD | 73 (61%) | 28 (62%) | 45 (61%) | 0.9 |
| Heart failure | 3 (2.5%) | 2 (4.4%) | 1 (1.4%) | 0.6 |
| Ischemic heart disease | 4 (3.4%) | 1 (2.2%) | 3 (4.1%) | >0.9 |
| Obesity | 17 (14%) | 7 (16%) | 10 (14%) | 0.8 |
| Oral candidiasis | 25 (21%) | 10 (22%) | 15 (20%) | 0.8 |
| Osteoporosis | 43 (36%) | 16 (36%) | 27 (36%) | >0.9 |
| OSAS | 6 (5.0%) | 1 (2.2%) | 5 (6.8%) | 0.4 |
| Data are presented as: n (%) or median (IQR), unless otherwise is stated. ^b^Based on ATC-codes for prescriptions from 1995 and onward ICD-10-codes for secondary care diagnoses 10 years prior to the index date. ICS: Inhaled corticosteroid; ABPA: Allergic bronchopulmonary aspergillosis. AERD: Aspirin-exacerbated respiratory disease; COPD: Chronic obstructive pulmonary disease; EGPA: Eosinophilic Granulomatosis with polyangiitis  GERD: Gastroesophageal reflux disease; OSAS: Obstructive sleep apnoea syndrome. ^a^Wilcoxon rank sum test, Pearson’s Chi-squared test or Fisher’s exact test used. | | | | |

| **Supplementary Table 3.** Changes in exposed and prescribed inhaled corticosteroid doses stratified by clinical treatment outcome to biologic therapy as  non-response, clinical response, and clinical remission in 361 patients with severe asthma | | | | | | | |
| --- | --- | --- | --- | --- | --- | --- | --- |
|  | | | **Overall**  **(n=361)** | **Non-response**  **(n=42)** | **Clinical response**  **(n=247)** | **Clinical remission**  **(n=72)** | **p-value^b^** |
| **Daily prescribed dose of ICS (ug)^a^** | | | | | | |  |
|  | At baseline | | 1600 (1600-2296) n=291 | 1600 (1100-1600) n=32 | 1600 (1600-2296) n=204 | 1600 (1548-2400) n=55 | 0.3 |
|  | At 12 months | | 1600 (1200-2000) n=290 | 1600 (1374-1600) n=31 | 1600 (1496-2000) n=201 | 1600 (800-2296) n=58 | 0.7 |
| **Daily exposed dose of ICS (ug)^a^** | | |  |  |  |  |  |
|  | At baseline | | 1266 (890-1714) n=291 | 1121 (626-1692) n=32 | 1266 (916-1712) n=204 | 1333 (943-1733) n=55 | 0.4 |
|  |  | Of which supratherapeutic | 103 (29%) n=361 | 12 (29%) n=42 | 69 (28%) n=247 | 22 (31%) n=72 | >0.9 |
|  | At 12 months | | 1262 (806-1710) n=291 | 1052 (624-1578) n=32 | 1262 (842-1710) n=204 | 1328 (947-1821) n=55 | 0.11 |
|  |  | Of which supratherapeutic | 103 (29%) n=361 | 9 (21%) n=42 | 74 (30%) n=247 | 20 (28%) n=72 | 0.5 |
| Data are presented as: n (%) or median (IQR), unless otherwise is stated. ICS: inhaled corticosteroid; ^b^*budesonide* equivalent dose, ^b^Kruskal-Wallis rank sum test or Pearson’s Chi-squared test used. | | | | | | | |
